# Evaluating associations of migraine‐related vertigo

**DOI:** 10.1111/head.15038

**Published:** 2025-10-26

**Authors:** Nazia Karsan, Nicolas Vandenbussche, Robyn Jenia‐Wilcha, Pubudu Amarasena, Pannathat Soontrapa, Karthik Nagaraj, Carlo Lastarria Perez, Peter J. Goadsby

**Affiliations:** ^1^ Headache Group, Wolfson Sensory, Pain and Regeneration Centre (SPaRC) Institute of Psychiatry, Psychology and Neuroscience King's College London London UK; ^2^ NIHR King's Clinical Research Facility and South London and Maudsley (SLAM) Biomedical Research Centre King's College Hospital London UK; ^3^ Department of Neurology Vallesur Auna Clinic Arequipa Peru; ^4^ Department of Neurology AZ Sint‐Jan Bruges Bruges Belgium; ^5^ Department of Basic and Applied Medical Sciences, Faculty of Medicine and Health Sciences Ghent University Ghent Belgium; ^6^ Division of Neurology, Department of Medicine, Faculty of Medicine Siriraj Hospital, Mahidol University Bangkok Thailand; ^7^ Department of Neurology Morriston Hospital, Swansea Bay University Health Board Swansea UK

**Keywords:** allodynia, aura, chronic migraine, migraine, vertigo

## Abstract

**Objective:**

We set out to examine detailed phenotypic data from our clinic for associations of vertigo in chronic migraine.

**Background:**

Vertigo is a non‐canonical, common symptom of migraine. Little is known about its associations with other symptoms within the migraine phenotype. There is significant methodological heterogeneity and therefore inadequate overall evidence about the potential differences in efficacy of any migraine treatments in patients with problematic vertigo associated with migraine, compared to those without vertigo. Enhancing understanding of migraine‐related vertigo could help guide treatment and inform on mechanisms of vestibular migraine, a poorly understood diagnostic entity.

**Methods:**

Chronic migraine extended phenotypes of patients seen within the adult headache service at King's College Hospital Tertiary Headache Service between January 2014 and December 2021 (*n* = 589) were extracted from the first documented clinic consultation letter retrospectively. For those with information about vertigo (*n* = 562), potential associations of interest for the presence of vertigo (gender, allodynia, aura, photophobia, phonophobia and osmophobia, baseline headache frequency, number of premonitory symptoms, presence of cranial autonomic symptoms, and age) were analyzed using a regression model (IBM SPSS v 29). Missing data were excluded (final *n* = 435).

**Results:**

The total sample size for analysis in the regression model was *n* = 435, after excluding missing data (*n* = 126) and outliers (*n* = 1). Patients were 16–92 years old (median, 47; interquartile range, 37–55), and the majority (83.4%) were female. Vertigo associated with migraine was present in 275 of 562 (49%) patients. Within the regression model, the presence of aura (odds ratio, 2.13; 95% confidence interval, 1.4–3.23, *P* < 0.001) and allodynia (odds ratio, 2.74; 95% confidence interval, 1.76–4.26, *P* < 0.001) were positively associated with vertigo.

**Conclusions:**

Vertigo in chronic migraine is common and may be associated with a more enriched phenotype independent of baseline headache frequency. Future treatment strategies should be evaluated for their effects on this often under‐recognized yet disabling symptom.

## Plain language summary

This study examined predictors of vertigo associated with chronic migraine in a large cohort of adults attending a specialist headache clinic in London, UK, using retrospective data collection from the first clinic consultation letter. The study found that vertigo associated with migraine occurs in 49% of patients; in the analysis, it was associated with the presence of allodynia (a sensitivity to touch over the head or neck) and the presence of aura. Targeting migraine beyond the headache itself may be useful in managing vertigo, and the combination of symptoms could be caused by the interaction of several brain pathways.

**Abbreviations**
CAS: cranial autonomic symptoms
ICHD: International Classification of Headache Disorders
PS: premonitory symptoms
VM: vestibular migraine

## INTRODUCTION

Migraine is a common and disabling brain disorder, which manifests as recurring attacks of headache associated with other symptoms like sensory sensitivities, nausea, and vomiting.^1^ Other non‐canonical symptoms associated with migraine, such as allodynia, vertigo, cognitive and emotional changes, and altered arousal, to name a few, are also well reported and contribute to a heterogeneous clinical phenotype, with significant intra‐ and inter‐patient variability.^2^

Vertigo associated with migraine is a common symptom^3^, ^4^, ^5^, ^6^, ^7^, ^8^, and has been reported as far back as the second century.^9^ However, over the last decade, it only features within the International Classification of Headache Disorders version 3 (ICHD‐3) in vestibular migraine (VM), as an appendix diagnosis, and in the main ICHD‐3 classification, in migraine with brainstem aura, which is a distinct and rare entity.^10^ VM is a clinical syndrome that aimed to encapsulate and merge previous terminologies such as “migraine‐associated vertigo, dizziness, or vestibulopathy.” In VM, vestibular symptoms are moderate or severe in intensity (in that they either interfere with, or cause cessation of daily activities).^1^ Overall, therefore, mild vertigo associated with most migraine is likely probably under‐recognized and under‐reported. Given the current structure of the ICHD‐3, VM is an appendix diagnosis and therefore should be applied if another diagnosis within the main classification does not better account for the clinical phenotype (as per criterion E).^1^ Up to 60% of those with chronic migraine are thought to fulfill VM criteria, and up to 73% of those with migraine with aura,^11^ yet the literature would suggest far lower prevalence of VM across different settings.^12^ The heterogeneity in what patients may call “vertigo” or “dizziness,”^13^ the way they are questioned about the co‐existence of such symptoms and headache, and the sometimes temporal dissociation between vertigo and headache in migraine can all further contribute to the under‐recognition of this important symptom.^12^ Akin to other symptoms in migraine, vertigo can also occur during different phases of the migraine attack, from the premonitory phase, aura, headache phase, through to following headache resolution in the postdrome.^4^ Interestingly, as well as the association of migraine with vertigo symptomatically, vestibular stimulation (with rotation or caloric testing) has been reported to trigger migraine,^14^, ^15^ suggesting a biological link between the vestibular and trigeminal systems.^16^ Importantly, headache frequency, severity, longer headache duration, depression, anxiety, and disability have all been reported to be positively correlated with vertigo,^17^ suggesting that, like allodynia, it may serve as a symptom biomarker of disease severity, chronicity, and increased disability^18^. Migraine therapeutics research should address the effects of therapies on such symptoms because they may represent markers of disease severity and perhaps predict poor response to therapy.

Other vestibular conditions such as Meniere's disease, motion sickness, and benign paroxysmal positional vertigo are reported to be comorbid with migraine, further suggesting shared neurobiology and neuro‐otological mechanisms between vertigo and headache, likely related to the interactions between the vestibular and trigeminal systems and sensory sensitization of thalamic and cortical brain structures.^19^ In particular, such mechanisms are feasibly relevant in migraine given the definition of vestibular symptoms by ICHD‐3^1^ and the Barany Society Classification.^20^ These include a sense of and sensitivity to motion, and include the effects of head position, head motion, visual stimulation, and the association with nausea. The themes of these provide links around altered sensory processing and sensitization with other features of migraine.^16^ VM might reflect sensitivity to vestibular stimulation, akin to the sensitivity to light and sound that patients with migraine commonly report.^21^

Furthering understanding of vertigo within the extended phenotype of migraine could provide neurobiological and therapeutic insights. For this study, we aimed to systematically study vertigo within the extended phenotype of chronic migraine, in order to explore its potential associations with other migraine‐related features. In particular, we were interested in symptoms that could also be associated with sensitization and altered sensory processing. We decided to study chronic migraine alone, given this is the most common disorder seen in the tertiary headache service, and the positive skew of headache days in our patient population. There were insufficient patients with episodic migraine in our practice to include in this study to allow for equal groups.

The primary hypothesis of the study was that vertigo is common in chronic migraine, and is associated with increased symptoms both within and outside of the canonical symptoms of migraine. This could include aura and allodynia, consistent with the previous literature.

## METHODS

The study was registered with the Neurology Department at King's College Hospital as a service evaluation of our detailed headache history taking, with the aim of quality improvement. The data were compiled and analyzed with this objective, and such a study does not require research ethics committee review in the United Kingdom (http://www.hra‐decisiontools.org.uk/research/). Informed consent was not required for use of patient data for this study because the data were acquired from notes and clinic letters for patients, and data collection was entirely anonymized, so only local hospital approval was needed.

**FIGURE 1:**
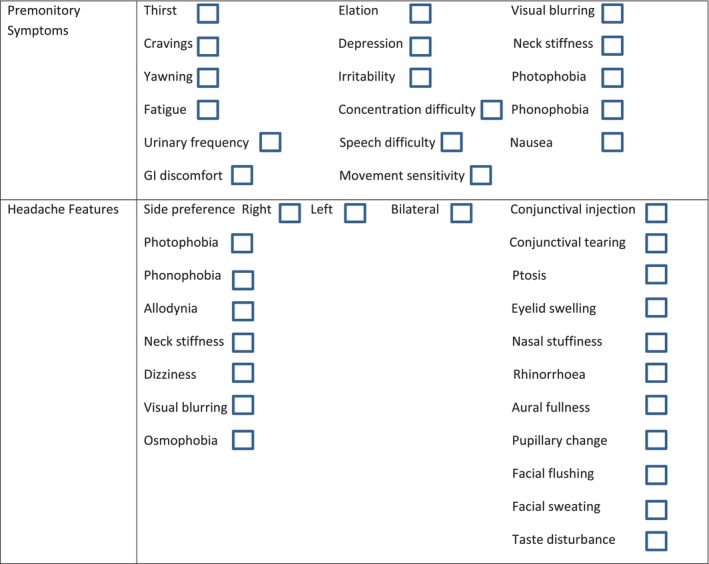
Physician‐administered symptom questionnaire used to collect patient data at the first clinic visit. [Color figure can be viewed at wileyonlinelibrary.com]

A retrospective search was performed for the words “chronic migraine” within first clinic letters of systematically phenotyped patients seen within the King's College Hospital Tertiary Headache Service between January 2015 and December 2021. All the selected patients had ICHD‐3 or ICHD‐3beta diagnosed chronic migraine (with or without aura). New daily persistent headache of any type, or superimposed other primary or secondary headache disorders were excluded. All the patients had undergone a detailed first appointment with a trained specialist headache physician during which a systematic extended headache history was taken to acquire the detailed clinical phenotype. This is typically performed using a symptom questionnaire that we have used before for this type of data capture in similar studies (Figure 1). This includes premonitory symptoms (PS), aura, headache‐associated symptoms (vertigo, allodynia, and osmophobia), and cranial autonomic symptoms (CAS). Despite various forms of dizziness being reported in the letters, these were all grouped into vertigo or light‐headedness, depending on if there was the perception of altered motion (present in the former only), or not. Vertigo and all migraine‐associated symptoms were coded, as binary variables (yes/no for presence/absence). Unfortunately, there was no systematic recording of vertigo duration, temporal association with headache, frequency of vertigo attacks, or temporal association between historical headache and vertigo onsets, as well as disability caused by vertigo, therefore, these data were not collected. For allodynia, patients were typically asked “With your migraine, is it more sensitive than usual to touch your head or neck?”

### Sample size calculation

Given this was an exploratory study looking at vertigo prevalence in chronic migraine across 6 years in our clinic, no specific statistical power calculation was conducted before the study. The sample size was based on the available data.

### Data collection

**FIGURE 2:**
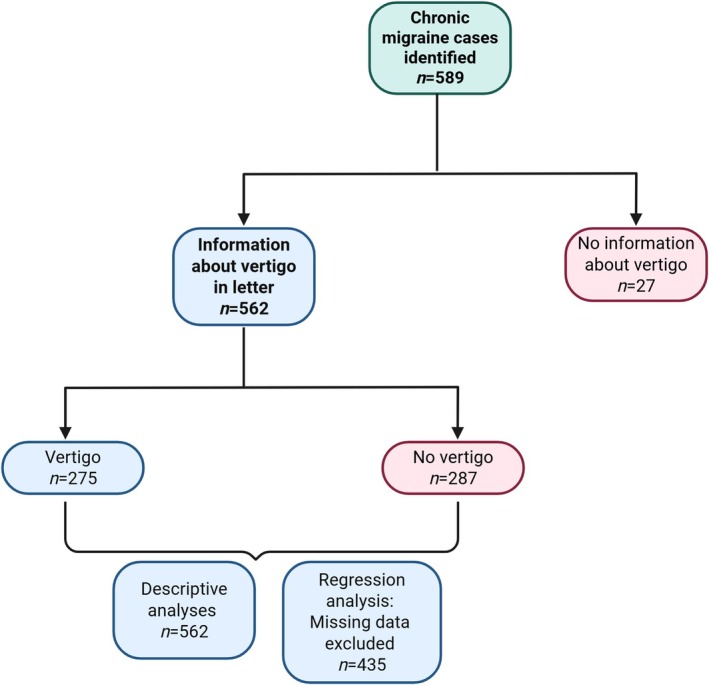
Flowchart of the patients at each stage of the study and in different analyses. [Color figure can be viewed at wileyonlinelibrary.com]

In total, 589 patients seen in the selected time frame were identified with chronic migraine as the final headache diagnosis. Of these, 27 patients had no clear information about vertigo and were excluded from the study. This left 562 patients with information about vertigo. Not all of these were included in the regression analysis (see Figure 2). All data were entered into an Excel spreadsheet and subsequently exported into IBM SPSS version 29 (IBM Corp., Armonk, NY, USA) for statistical analysis. The data were acquired retrospectively from patient reporting at the first clinical encounter.

### Data analysis

The total sample size for the regression model after exclusion of missing data (*n* = 127) was *n* = 435.

#### Descriptive statistics

Descriptive statistics were used to summarize and describe the key characteristics of the wider patient cohort with chronic migraine (*n* = 562). Mean and standard deviation (SD) were used for the age information (as the ages of the cohort were normally distributed). Median and interquartile ranges were used for headache days per month given the skew toward more headache days in this patient cohort. Counts with percentages were used for categorical data.

A binomial logistic regression was performed to ascertain the effects of age, sex, presence of aura, monthly headache days (/30), presence of photophobia, phonophobia, osmophobia, allodynia, CAS, and PS on the likelihood of reporting vertigo. Linearity of the continuous variables (age and headache days per month) with respect to the logit of the dependent variable (vertigo presence) was assessed via the Box‐Tidwell (1962) procedure. Collinearity of the categorical variables was assessed by generating variance inflation factors, all of which were between 1.1 and 1.2, suggesting unlikely multicollinearity. A Bonferroni correction was applied using all 13 terms in the model, resulting in statistical significance being accepted when *P* < 0.004^52^. We therefore used two‐sided hypothesis tests with an initial threshold of *P* < 0.05 and a Bonferroni correction was applied, so statistical significance was accepted at *P* < 0.004. Based on this assessment, all the continuous independent variables were found to be linearly related to the logit of the dependent variable. There was one case with a standardized residual of 2.1 SDs, which was removed from the analysis. The logistic regression model was statistically significant (χ^2^ [12] = 54.40, *P* < 0.001). The model explained 16% of the variance in vertigo reporting (Nagelkerke *R* ^2^), and correctly classified 65% of cases. The Hosmer and Lemeshow test was not significant (χ^2^ [8] = 6.21, *P* = 0.62), suggesting a good model fit. The addition of the independent variables improved the overall prediction of cases into the observed categories of the dependent variable from 50% to 64% (percentage accuracy in classification). Examination of a correlation matrix suggested no collinearity between age and headache days per month (the two continuous variables; correlation coefficient, 0.1).

The frequency counts of missing data were as follows; age (6), sex (1), aura (3), headache days per month (7), phonophobia (9), phonophobia (9), osmophobia (12), allodynia (11), CAS (110), and PS (31) (see Table 1).

**TABLE 1:** Summary of patient demographics and headache phenotypes of the patient cohort (*n* = 562).

	Whole chronic migraine cohort (*n* = 562)	No. of patients for whom information was available	With vertigo (*n* = 275)	Without vertigo (*n* = 287)
Age, range in years; mean (SD)	18–92; 47.5 (15.1)	556	16–83; 46.8 (14.4)	17–92; 47.6 (15.5)
Female sex, *n* (%)	489 (83.4)	561	234 (85.1)	237 (82.6)
Headache days per month, range in days, median (IQR)	15–30; 30 (22–30)	555	15–30; 30 (22–30)	15–30; 30 (23–30)
Aura, yes, *n* (%)	297 (51.1)	559	171 (62.4)	121 (42.5)
Allodynia, yes, *n* (%)	358 (64.6)	551	209 (76.6)	148 (53.2)
Photophobia, yes, *n* (%)	501 (90.1)	553	247 (90.5)	252 (90)
Phonophobia, yes, *n* (%)	481 (86.5)	553	242 (88.6)	237 (84.6)
Osmophobia, yes, *n* (%)	320 (57.9)	550	174 (64.2)	144 (51.6)
PS, yes, *n* (%)	472 (88.1)	531	245 (93.2)	223 (83.2)
CAS, yes, *n* (%)	347 (76.4)	452	182 (81.3)	163 (71.5)

Abbreviations: CAS, cranial autonomic symptoms; IQR, interquartile range; PS, premonitory symptoms; SD, standard deviation.

Within the regression model, 127 cases were excluded due to missing data and the exclusion of one outlier.

## RESULTS

This is the primary analysis of these data.

### Patient demographics

In total, 589 patients seen in the selected time frame were identified with chronic migraine as the final headache diagnosis. Of these, 27 patients had no clear information about vertigo and were excluded from the study. This left 562 patients with information about vertigo. Not all of these were included in the regression analysis, however, demographic and descriptive data are summarized for all these patients. The demographics of the patients are summarized in Table 1.

The age range of patients was 16–92 years (mean, 47.5 years, SD, 15.1), and the majority (83.4%) were female.

Over half of the patients (51.1%) reported aura associated with migraine attacks, the most common being visual, followed by sensory. This is more than would be expected and likely related to the tertiary headache clinic sampling. All the patients with migraine with aura reported a combination of attacks with and without aura.

The median number of headache days was 30 (range, 15–30 days; interquartile range, 22–30).

The headache diagnoses were all chronic migraine with (51.1%) and without aura (48.9%), and no patients had a formal VM diagnosis.

### Vertigo reporting

Almost half of the patients (275 of 562; 48.9%) overall reported vertigo associated with migraine. Table 1 shows the demographic and headache phenotype differences between the patients with and without vertigo.

### Other migraine‐associated symptoms

The frequencies of reporting of other migraine‐associated symptoms are shown in Table 1. Reporting of at least one PS was common overall (88.1%), the most common being concentration difficulty, mood change, and neck discomfort (Table 2). Similarly, the reporting of at least CAS was present in 76.4%, the most common being facial changes like flushing or pallor, lacrimation, and nasal congestion (Table 3). Allodynia was reported in 64.6%.

**TABLE 2:** Percentage of patients reporting each PS and the 95% Confidence Interval (CI) of the mean.

Premonitory symptom	Percentage reporting the symptom	95% Confidence Interval (CI)
Cognitive change	68	64–72
Mood change	59	55–63
Neck discomfort	45	41–49
Food cravings	24	20–28
Fatigue	38	34–42
Yawning	34	29–38
Gastrointestinal symptoms	21	18–25
Thirst	10	8–13

Abbreviations: CI, confidence interval; PS, premonitory symptom.

**TABLE 3:** Percentage of patients reporting each CAS and the 95% Confidence Interval (CI) of the mean.

Cranial autonomic symptom	Percentage reporting the symptom	95% Confidence Interval (CI)
Lacrimation	36	31–40
Facial changes (flushing or pallor)	38	33–42
Nasal congestion	33	28–37
Aural fullness	29	25–34
Conjunctival injection	26	22–30
Rhinorrhea	22	19–26
Pupillary change	18	14–21
Periorbital edema	16	12–19

Abbreviations: CAS, cranial autonomic symptom; CI, confidence interval.

### Regression model

#### Associations of vertigo

In the regression analysis, 435 patients were included. Of the 10 independent variables in the regression model, two were statistically significant following Bonferroni correction: presence of aura and allodynia (see Table 4). Those with baseline aura had 2.13 times higher odds of reporting vertigo (95% CI for adjusted odds ratio, 1.40–3.23; *P* < 0.001) and those with allodynia 2.74 times higher odds of reporting vertigo (95% CI for adjusted odds ratio, 1.76–2.26, *P* < 0.001) (see Tables 4 and 5).

**TABLE 4:** Binomial logistic univariate regression looking at the associations of vertigo with each variable separately without controlling for the other variables in the model (crude or unadjusted ORs are shown).

Variable	Odds Ratio (OR)	95% Confidence Interval (CI), lower–upper	*P*
Age	0.10	0.99–1.01	0.562
Female sex	1.18	0.75–1.9	0.473
Aura	2.25	1.60–3.16	**<0.001**
Monthly headache days	0.99	0.96–1.02	0.412
Phonophobia	1.42	0.86–2.32	0.168
Photophobia	1.06	0.60–1.85	0.850
Osmophobia	1.68	1.20–2.37	**0.003**
Allodynia	2.87	1.99–4.14	**<0.001**
Presence of CAS	1.73	1.11–2.69	0.015
Presence of PS	2.75	1.54–4.89	**<0.001**

*Note*: *P* values of variables with statistical significance are shown in bold.

Abbreviations: CAS, cranial autonomic symptom; CI, confidence interval; OR, odds ratio; PS, premonitory symptom.

**TABLE 5:** Binomial logistic regression (multivariate) looking at the associations of vertigo for each variable while accounting for all the variables in the model (adjusted OR, aOR are shown).

Variable	Adjusted Odds Ratio (aOR)	95% Confidence Interval (CI), lower–upper	*P*
Age	0.97	0.71–1.34	0.870
Female sex	1.00	0.56–1.80	0.993
Aura	2.13	1.40–3.23	**<0.001**
Monthly headache days	0.89	0.45–1.78	0.746
Phonophobia	1.15	0.60–2.23	0.673
Photophobia	0.66	0.30–1.44	0.298
Osmophobia	1.13	0.73–1.76	0.584
Allodynia	2.74	1.76–2.26	**<0.001**
Presence of CAS	1.32	0.80–2.16	0.279
Presence of PS	2.30	1.20–4.40	0.012

*Note*: *P* values of variables with statistical significance are shown in bold.

Abbreviations: aOR, adjusted odds ratio; CAS, cranial autonomic symptom; CI confidence interval; PS, premonitory symptom.

## DISCUSSION

We present here a large tertiary clinic study of adults looking at chronic migraine headache phenotypes, with regard to predictors of vertigo. We find that aura and allodynia are positively associated with vertigo. The reporting of aura is higher than what would be expected in the general population and this is likely related to the tertiary headache clinic sampling. Similarly, the median headache days per month was 30 (daily headache), again because of the skewed chronic migraine population being seen within tertiary headache services.

It is interesting that although almost half of the patients reported vertigo associated with migraine, none of them had a formal VM diagnosis, yet all had a chronic migraine diagnosis. In our service, we tend to make a headache diagnosis from the main ICHD‐3 classification where it applies,^1^ and we reserve a VM diagnosis for patients in whom vertigo is the most troublesome symptom, rather than headache. In general, patients of this kind tend to be seen in audiovestibular and neuro‐otology clinics rather than in the specialist headache clinic. In addition, given we did not acquire data on the duration, true severity, and temporal association of vertigo and headache, it is not possible to know how many of these patients may meet diagnostic criteria for VM in retrospect. Going forward, we are asking patients about their most troublesome symptom within their migraine phenotype to better understand if this helps differentiate patients biologically and with regard to treatment choice and response.

The suggestion of vertigo being associated with a more enriched phenotype is perhaps unsurprising, but it is interesting that aside from allodynia, other sensory sensitivities like photophobia, phonophobia, and osmophobia do not seem to have an association with vertigo. In previous studies in children, osmophobia has been demonstrated to associate with allodynia, as well as increased migraine severity.^22^, ^23^ The strong positive relationship with allodynia is supportive of allodynia and vertigo being independent predictors of a more enriched migraine phenotype, and perhaps markers of increased migraine‐related disability, although this was not specifically examined here. The co‐association of allodynia and aura with vertigo has been suggested before in patients with VM when compared to episodic migraine.^24^ There is a supportive study suggesting that vertigo was twice as common in patients with migraine with aura compared to without.^17^

The association of vertigo with other multisensory brain functions has been suggested as a means to control body motion in space such as motion perception, spatial orientation, visuospatial attention, and spatial awareness,^25^ all of which have been reported as being impaired in migraine.^11^, ^26^, ^27^, ^28^, ^29^, ^30^ This integration of multisensory brain functions leads to overdependence on one when another has an abnormality, and this may be the reason why patients with vestibular issues tend to be over‐reliant on visual cues,^31^, ^32^, ^33^, ^34^ and encounter more problematic vertigo in settings with high levels of visual stimulation. This is also seen in patients with VM, suggesting similar central mechanisms of motion and visuospatial orientation control. In addition to these findings, and the finding that vestibular stimulation can trigger migraine attacks, in experimental work, nitroglycerin, a potent migraine trigger, also has the ability to trigger vertigo associated with migraine,^2^ supporting the central role of sensory processing and integration in migraine, with subsequent sensitization.^16^ Increased motion sickness sensitivity^35^, ^36^ and travel sickness being a possible early marker of migraine in children^37^, ^38^, ^39^, ^40^ are likely mediated in a similar way through the abnormal interaction of visual and vestibular cues in some patients with migraine. Abnormal thresholds in motion detection in VM^41^ and altered spatial orientation during head tilt^42^ have been demonstrated experimentally, and suggest that the abnormal perception and awareness of body position and motion in VM may be caused by a dysfunction of multisensory processing and integration, namely between vestibular and visual stimuli.^21^

If migraine (not just VM) is taken more broadly, we know from functional imaging studies that altered brainstem^43^ and thalamocortical activity and function^44^ are likely to be involved in the neurobiology, including in the mediation of non‐painful symptoms of migraine like PS. There is therefore neurobiological plausibility for the additional interaction of the pathways controlling visuospatial function for example, with those that are involved in mediating visual aura, or that thalamic sensitization causing allodynia,^45^, ^46^, ^47^ may also be involved in vertigo.

Asking about vertigo associated with migraine and about the most bothersome symptom of the migraine attack may help characterize the prevalence of this symptom or of VM, and if present, may allude to a more enriched migraine phenotype. It is clear that for some patients, headache is not the most troublesome part of their migraine,^48^ and the primary outcome measure of headache days in treatment trials is often inadequate in capturing symptom control and disability, so the most bothersome symptom is an important co‐endpoint in clinical trials.^49^, ^50^, ^51^ Understanding what is the most disabling symptom for a patient may help guide future management, and allow individualization of therapy based on the presence of aura or vertigo for example (that may respond better to some drugs than conventionally used migraine prevention). Working together with audiovestibular and neuro‐otological clinics to identify patients with VM (presumably in whom vertigo is the most troublesome symptom) would help with these evaluations, and we are actively engaged in such collaborative efforts.

### Other findings

As well as the primary outcome of the predictors of vertigo, this study also found high frequencies of the reporting of PS, CAS, and allodynia in adults with chronic migraine.

### Limitations

This study demonstrates the predictors of vertigo in a large patient cohort from a tertiary headache clinic. The data collection was retrospective rather than prospective, which would have been ideal, but we decided to use the large sample size initially in this study to help to answer some important questions. Hopefully, a study of this kind using prospective data collection can be conducted in the future.

The exclusion rate of patients from the regression model due to missing data must be considered because this could potentially bias results if these patients were outliers. Given the retrospective design of the study and different clinicians acquiring the data at the first clinic encounter, this was difficult to control for. Going forward, we hope to be more systematic and consistent with the acquisition of this type of information, and we hope that given the large sample size, any significant results are translatable to chronic migraine overall.

The regression did not account for variables such as disease duration and headache severity and the presence of comorbid psychiatric disorders, which could feasibly be relevant confounders with regard to the reporting of vertigo. Their absence could therefore affect the fit and outcome of the regression model. We hope to repeat such a study in the future using systematic and consistent data collection at the first clinic encounter, to be able to assess for the effect of these on the outcomes. We hope that we can perform future, ideally, prospective studies gathering standardized vertigo metrics, depression and anxiety scores, headache severity and disease duration, vertigo duration, temporal occurrence in the course of the migraine attack, and more details on the phenotype of vertigo to help characterize the symptom further. Future prospective and systematic studies are needed to better characterize the population prevalence of vertigo in migraine.

## AUTHOR CONTRIBUTIONS

**Nazia Karsan:** Conceptualization; data curation; formal analysis; investigation; methodology; project administration; resources; validation; visualization; writing – original draft. **Nicolas Vandenbussche:** Conceptualization; data curation; formal analysis; investigation; methodology; project administration; resources; validation; visualization; writing – original draft. **Robyn Jenia‐Wilcha:** Data curation; methodology; project administration; resources. **Pubudu Amarasena:** Data curation; methodology; project administration; resources. **Pannathat Soontrapa:** Data curation; methodology; project administration; resources. **Karthik Nagaraj:** Conceptualization; data curation; investigation; methodology; project administration; resources. **Carlo Lastarria Perez:** Data curation; methodology; project administration; resources. **Peter J. Goadsby:** Conceptualization; data curation; supervision; writing – review and editing.

## FUNDING INFORMATION

The work has been supported in part by the NIHR SLaM Biomedical Research Centre (NIHR203318).

## CONFLICT OF INTEREST STATEMENT

**Nicolas Vandenbussche, Robyn Jenia‐Wilcha, Pubudu Amarasena, Pannathat Soontrapa, Karthik Nagaraj**, and **Carlo Lastarria Perez** declare no conflicts for interest. Unrelated to the submission, **Nazia Karsan** reports, over the last 36 months, personal fees for consulting from Pfizer, AbbVie and Teva Pharmaceuticals, travel support grants from Teva Pharmaceuticals, and publishing royalties or fees from Wolters Kluwer. **Peter J. Goadsby** reports, over the last 36 months, personal fees for consulting from Eon Biopharma, AbbVie, Aurene, CoolTech LLC, Dr Reddy's, Eli‐Lilly and Company, Epalex, Kallyope, Linpharma, Lundbeck, Orion Pharma, PureTech Health LLC, Satsuma, Shiratronics, Teva Pharmaceuticals, and Vial, and personal fees for advice through Gerson Lehrman Group, Guidepoint, SAI Med Partners, Vector Metric, and fees for educational materials from CME Outfitters and WebMD, and publishing royalties or fees from Massachusetts Medical Society, Oxford University Press, UptoDate and Wolters Kluwer.
